# Methyl 6-chloro-2-methyl-4-phenyl­quinoline-3-carboxyl­ate

**DOI:** 10.1107/S1600536809052891

**Published:** 2009-12-16

**Authors:** J. Kalyana Sundar, S. Natarajan, S. Sarveswari, V. Vijayakumar, J. Suresh, P. L. Nilantha Lakshman

**Affiliations:** a Department of Physics, Madurai Kamaraj University, Madurai 625 021, India; b Organic Chemistry Division, School of Advanced Sciences, VIT University, Vellore 632 014, India; c Department of Physics, The Madura College, Madurai 625 011, India; d Department of Food Science and Technology, Faculty of Agriculture, University of Ruhuna, Mapalana, Kamburupitiya 81100, Sri Lanka

## Abstract

In the title compound, C_18_H_14_ClNO_2_, the quinoline ring system is planar (r.m.s. deviation = 0.032 Å) and the phenyl ring is twisted away from it by 57.5 (1)°. The crystal structure is stabilized by weak C—H⋯π inter­actions.

## Related literature

For the anti-tuberculosis activity of quinoline-2-carboxylic acid derivatives, see: Jain *et al.* (2005 ▶).
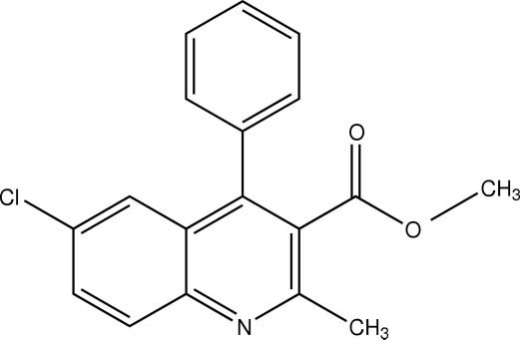


## Experimental

### 

#### Crystal data

C_18_H_14_ClNO_2_
*M* *_r_* = 311.75
Monoclinic, 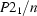

*a* = 10.828 (5) Å
*b* = 7.535 (4) Å
*c* = 18.829 (5) Å
β = 94.369 (5)°
*V* = 1531.8 (12) Å^3^
*Z* = 4
Mo *K*α radiation
μ = 0.26 mm^−1^
*T* = 293 K
0.18 × 0.16 × 0.13 mm

#### Data collection

Nonius MACH-3 diffractometer
Absorption correction: ψ scan (North *et al.*, 1968 ▶) *T*_min_ = 0.955, *T*_max_ = 0.967
3320 measured reflections
2682 independent reflections
2309 reflections with *I* > 2σ(*I*)
*R*_int_ = 0.018
2 standard reflections every 2 min
intensity decay: none

#### Refinement

*R*[*F* ^2^ > 2σ(*F* ^2^)] = 0.034
*wR*(*F* ^2^) = 0.100
*S* = 1.03
2682 reflections
202 parameters
H-atom parameters constrained
Δρ_max_ = 0.20 e Å^−3^
Δρ_min_ = −0.29 e Å^−3^

### 

Data collection: *CAD-4 EXPRESS* (Enraf–Nonius, 1994 ▶); cell refinement: *CAD-4 EXPRESS*; data reduction: *XCAD4* (Harms & Wocadlo, 1996 ▶); program(s) used to solve structure: *SHELXS97* (Sheldrick, 2008 ▶); program(s) used to refine structure: *SHELXL97* (Sheldrick, 2008 ▶); molecular graphics: *PLATON* (Spek, 2009 ▶); software used to prepare material for publication: *SHELXL97*.

## Supplementary Material

Crystal structure: contains datablocks global, I. DOI: 10.1107/S1600536809052891/ci2981sup1.cif

Structure factors: contains datablocks I. DOI: 10.1107/S1600536809052891/ci2981Isup2.hkl

Additional supplementary materials:  crystallographic information; 3D view; checkCIF report

## Figures and Tables

**Table 1:** Hydrogen-bond geometry (Å, °)

*D*—H⋯*A*	*D*—H	H⋯*A*	*D*⋯*A*	*D*—H⋯*A*
C9—H9*B*⋯*Cg*1^i^	0.96	2.80	3.744 (3)	166

Symmetry code: (i) 
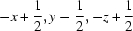
. *Cg*1 is the centroid of the C14–C19 ring.

**Fig. 1.**
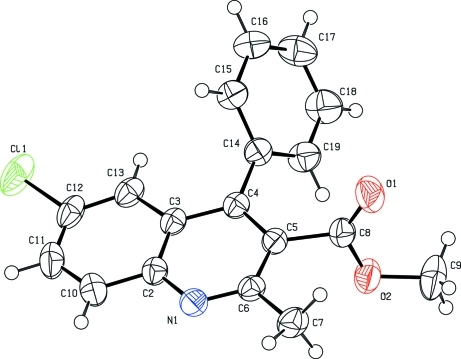
The molecular structure of the title compound, showing 50% probability displacement ellipsoids and the atom-numbering scheme.

## supplementary crystallographic information

### Comment

Quinoline-2-carboxylic acid derivatives are a class of important materials useful as anti-tuberculosis agents (Jain *et al.*, 2005). We report here the crystal structure of the title compound, a quinoline derivative.

The molecular structure of the title compound is shown in Fig.1. The quinoline ring system is planar (r.m.s. deviation is 0.032 Å). Due to phenyl substitution in the pyridine ring, the C4—C5 bond is longer [1.373 (2) Å] and the C3—C4 bond is shorter [1.434 (2) Å] than standard values for C═C (1.334 Å) and C*_sp_*^2^—C*_sp_*^2^ (1.455 Å) bond lengths respectively. The phenyl ring is twisted out of the quinoline ring system by 57.5 (1)°. A weak C—H···π interaction involving the C14–C19 ring is observed.

### Experimental

A mixture of 2-amino-5-chlorobenzophenone (2.3 g, 0.01 mol) and methyacetoacetate (1.2 g, 0.01 mmol) with 0.15 ml concentrated HCl taken in a beaker was subjected to microwave irradiation for about 6 min. After completion of the reaction (TLC), the reaction mixture was washed with saturated NaHCO_3_ solution (10 ml), dried, washed with petroleum ether and recrystallized with chloroform (m.p. 134–135 °C).

### Refinement

H atoms were placed at calculated positions and allowed to ride on their carrier atoms with C–H = 0.93–0.96 Å and *U*_iso_ = 1.2*U*_eq_(C) for CH groups, and 1.5*U*_eq_ for CH_3_ groups.

### Crystal data

**Table:**

C_18_H_14_ClNO_2_	*F*(000) = 648
*M**_r_* = 311.75	*D*_x_ = 1.352 Mg m^−^^3^
Monoclinic, *P*2_1_/*n*	Mo *K*α radiation, λ = 0.71069 Å
Hall symbol: -P 2yn	Cell parameters from 25 reflections
*a* = 10.828 (5) Å	θ = 2–25°
*b* = 7.535 (4) Å	µ = 0.26 mm^−^^1^
*c* = 18.829 (5) Å	*T* = 293 K
β = 94.369 (5)°	Block, colourless
*V* = 1531.8 (12) Å^3^	0.18 × 0.16 × 0.13 mm
*Z* = 4	

### Data collection

**Table:**

Nonius MACH-3 diffractometer	2309 reflections with *I* > 2σ(*I*)
Radiation source: fine-focus sealed tube	*R*_int_ = 0.018
graphite	θ_max_ = 25.0°, θ_min_ = 2.1°
ω–2θ scans	*h* = 0→12
Absorption correction: ψ scan (North *et al.*, 1968)	*k* = −1→8
*T*_min_ = 0.955, *T*_max_ = 0.967	*l* = −22→22
3320 measured reflections	2 standard reflections every 60 min
2682 independent reflections	intensity decay: none

### Refinement

**Table:**

Refinement on *F*^2^	Secondary atom site location: difference Fourier map
Least-squares matrix: full	Hydrogen site location: inferred from neighbouring sites
*R*[*F*^2^ > 2σ(*F*^2^)] = 0.034	H-atom parameters constrained
*wR*(*F*^2^) = 0.100	*w* = 1/[σ^2^(*F*_o_^2^) + (0.0502*P*)^2^ + 0.5537*P*] where *P* = (*F*_o_^2^ + 2*F*_c_^2^)/3
*S* = 1.03	(Δ/σ)_max_ = 0.001
2682 reflections	Δρ_max_ = 0.20 e Å^−^^3^
202 parameters	Δρ_min_ = −0.29 e Å^−^^3^
0 restraints	Extinction correction: *SHELXL97* (Sheldrick, 2008), Fc^*^=kFc[1+0.001xFc^2^λ^3^/sin(2θ)]^-1/4^
Primary atom site location: structure-invariant direct methods	Extinction coefficient: 0.0132 (14)

### Special details

**Table:**

Geometry. All e.s.d.'s (except the e.s.d. in the dihedral angle between two l.s. planes) are estimated using the full covariance matrix. The cell e.s.d.'s are taken into account individually in the estimation of e.s.d.'s in distances, angles and torsion angles; correlations between e.s.d.'s in cell parameters are only used when they are defined by crystal symmetry. An approximate (isotropic) treatment of cell e.s.d.'s is used for estimating e.s.d.'s involving l.s. planes.
Refinement. Refinement of *F*^2^ against ALL reflections. The weighted *R*-factor *wR* and goodness of fit *S* are based on *F*^2^, conventional *R*-factors *R* are based on *F*, with *F* set to zero for negative *F*^2^. The threshold expression of *F*^2^ > σ(*F*^2^) is used only for calculating *R*-factors(gt) *etc*. and is not relevant to the choice of reflections for refinement. *R*-factors based on *F*^2^ are statistically about twice as large as those based on *F*, and *R*- factors based on ALL data will be even larger.

### Fractional atomic coordinates and isotropic or equivalent isotropic displacement parameters (Å^2^)

**Table:**

	*x*	*y*	*z*	*U*_iso_*/*U*_eq_	
C2	0.46859 (14)	0.2554 (2)	−0.02447 (8)	0.0362 (3)	
C3	0.53086 (13)	0.25646 (19)	0.04444 (8)	0.0346 (3)	
C4	0.46120 (13)	0.30371 (19)	0.10339 (8)	0.0339 (3)	
C5	0.33707 (13)	0.33626 (19)	0.08882 (8)	0.0340 (3)	
C6	0.28365 (13)	0.3408 (2)	0.01735 (8)	0.0358 (3)	
C7	0.15150 (15)	0.3971 (3)	0.00113 (10)	0.0487 (4)	
H7A	0.1384	0.4275	−0.0484	0.073*	
H7B	0.1343	0.4985	0.0297	0.073*	
H7C	0.0973	0.3015	0.0117	0.073*	
C8	0.25152 (14)	0.3699 (2)	0.14669 (8)	0.0410 (4)	
C9	0.0887 (2)	0.2382 (3)	0.20546 (13)	0.0727 (6)	
H9A	0.1254	0.2793	0.2505	0.109*	
H9B	0.0525	0.1233	0.2114	0.109*	
H9C	0.0257	0.3200	0.1878	0.109*	
C10	0.53354 (17)	0.2038 (2)	−0.08359 (9)	0.0485 (4)	
H10	0.4928	0.2031	−0.1289	0.058*	
C11	0.65448 (17)	0.1555 (2)	−0.07501 (10)	0.0529 (5)	
H11	0.6968	0.1224	−0.1141	0.063*	
C12	0.71432 (14)	0.1561 (2)	−0.00690 (11)	0.0472 (4)	
C13	0.65654 (14)	0.2040 (2)	0.05190 (9)	0.0427 (4)	
H13	0.6994	0.2022	0.0966	0.051*	
C14	0.52259 (14)	0.3155 (2)	0.17690 (8)	0.0386 (4)	
C15	0.62527 (15)	0.4262 (2)	0.19060 (9)	0.0451 (4)	
H15	0.6545	0.4927	0.1538	0.054*	
C16	0.68357 (17)	0.4376 (3)	0.25823 (10)	0.0548 (5)	
H16	0.7522	0.5109	0.2667	0.066*	
C17	0.6404 (2)	0.3406 (3)	0.31331 (10)	0.0608 (5)	
H17	0.6805	0.3469	0.3587	0.073*	
C18	0.5376 (2)	0.2341 (3)	0.30062 (10)	0.0608 (5)	
H18	0.5072	0.1710	0.3380	0.073*	
C19	0.47923 (17)	0.2203 (2)	0.23292 (9)	0.0489 (4)	
H19	0.4106	0.1467	0.2249	0.059*	
N1	0.34737 (12)	0.30270 (18)	−0.03732 (7)	0.0394 (3)	
O1	0.24231 (13)	0.50480 (19)	0.17883 (8)	0.0677 (4)	
O2	0.18315 (11)	0.22595 (16)	0.15517 (7)	0.0536 (3)	
Cl1	0.86967 (4)	0.09440 (7)	0.00332 (4)	0.0739 (2)	

### Atomic displacement parameters (Å^2^)

**Table:**

	*U*^11^	*U*^22^	*U*^33^	*U*^12^	*U*^13^	*U*^23^
C2	0.0368 (8)	0.0318 (8)	0.0408 (8)	0.0000 (6)	0.0077 (6)	0.0017 (6)
C3	0.0317 (7)	0.0288 (7)	0.0439 (8)	0.0001 (6)	0.0060 (6)	0.0023 (6)
C4	0.0340 (7)	0.0294 (7)	0.0384 (8)	−0.0006 (6)	0.0036 (6)	0.0018 (6)
C5	0.0322 (7)	0.0302 (7)	0.0398 (8)	0.0004 (6)	0.0048 (6)	−0.0011 (6)
C6	0.0322 (7)	0.0312 (8)	0.0439 (8)	−0.0005 (6)	0.0021 (6)	−0.0014 (6)
C7	0.0339 (8)	0.0538 (11)	0.0572 (10)	0.0064 (7)	−0.0029 (7)	−0.0010 (8)
C8	0.0369 (8)	0.0427 (9)	0.0440 (9)	0.0030 (7)	0.0071 (6)	−0.0028 (7)
C9	0.0644 (13)	0.0759 (15)	0.0835 (15)	0.0002 (11)	0.0421 (11)	0.0110 (12)
C10	0.0536 (10)	0.0489 (10)	0.0445 (9)	0.0026 (8)	0.0140 (7)	−0.0021 (8)
C11	0.0532 (10)	0.0462 (10)	0.0629 (11)	0.0032 (8)	0.0287 (9)	−0.0036 (8)
C12	0.0329 (8)	0.0321 (8)	0.0786 (12)	0.0026 (6)	0.0173 (8)	0.0004 (8)
C13	0.0332 (8)	0.0362 (8)	0.0588 (10)	0.0016 (6)	0.0042 (7)	0.0026 (7)
C14	0.0359 (8)	0.0393 (9)	0.0402 (8)	0.0030 (6)	0.0005 (6)	0.0016 (7)
C15	0.0432 (9)	0.0459 (9)	0.0456 (9)	−0.0035 (7)	0.0002 (7)	0.0039 (7)
C16	0.0505 (10)	0.0550 (11)	0.0568 (10)	−0.0077 (8)	−0.0097 (8)	−0.0015 (9)
C17	0.0713 (13)	0.0643 (12)	0.0440 (10)	−0.0029 (10)	−0.0143 (9)	0.0021 (9)
C18	0.0739 (13)	0.0627 (12)	0.0445 (10)	−0.0086 (10)	−0.0030 (9)	0.0138 (9)
C19	0.0511 (10)	0.0488 (10)	0.0461 (9)	−0.0072 (8)	−0.0004 (7)	0.0076 (8)
N1	0.0374 (7)	0.0410 (7)	0.0396 (7)	0.0020 (6)	0.0013 (5)	−0.0003 (6)
O1	0.0707 (9)	0.0562 (8)	0.0804 (10)	−0.0041 (7)	0.0339 (7)	−0.0240 (7)
O2	0.0524 (7)	0.0487 (7)	0.0629 (8)	−0.0063 (6)	0.0258 (6)	−0.0024 (6)
Cl1	0.0349 (3)	0.0598 (3)	0.1295 (5)	0.0111 (2)	0.0227 (3)	−0.0040 (3)

### Geometric parameters (Å, °)

**Table:**

C2—N1	1.364 (2)	C9—H9C	0.96
C2—C10	1.416 (2)	C10—C11	1.357 (3)
C2—C3	1.416 (2)	C10—H10	0.93
C3—C13	1.414 (2)	C11—C12	1.392 (3)
C3—C4	1.434 (2)	C11—H11	0.93
C4—C5	1.373 (2)	C12—C13	1.361 (2)
C4—C14	1.492 (2)	C12—Cl1	1.7418 (16)
C5—C6	1.424 (2)	C13—H13	0.93
C5—C8	1.505 (2)	C14—C19	1.387 (2)
C6—N1	1.3139 (19)	C14—C15	1.398 (2)
C6—C7	1.501 (2)	C15—C16	1.380 (2)
C7—H7A	0.96	C15—H15	0.93
C7—H7B	0.96	C16—C17	1.379 (3)
C7—H7C	0.96	C16—H16	0.93
C8—O1	1.191 (2)	C17—C18	1.378 (3)
C8—O2	1.330 (2)	C17—H17	0.93
C9—O2	1.448 (2)	C18—C19	1.383 (3)
C9—H9A	0.96	C18—H18	0.93
C9—H9B	0.96	C19—H19	0.93
			
N1—C2—C10	117.46 (14)	C11—C10—H10	119.6
N1—C2—C3	123.08 (13)	C2—C10—H10	119.6
C10—C2—C3	119.45 (14)	C10—C11—C12	119.08 (15)
C13—C3—C2	118.49 (14)	C10—C11—H11	120.5
C13—C3—C4	123.48 (14)	C12—C11—H11	120.5
C2—C3—C4	117.97 (13)	C13—C12—C11	122.66 (15)
C5—C4—C3	117.02 (13)	C13—C12—Cl1	118.74 (15)
C5—C4—C14	122.34 (13)	C11—C12—Cl1	118.60 (13)
C3—C4—C14	120.64 (13)	C12—C13—C3	119.47 (16)
C4—C5—C6	120.93 (13)	C12—C13—H13	120.3
C4—C5—C8	122.19 (13)	C3—C13—H13	120.3
C6—C5—C8	116.88 (13)	C19—C14—C15	118.62 (15)
N1—C6—C5	122.37 (13)	C19—C14—C4	121.47 (14)
N1—C6—C7	116.85 (14)	C15—C14—C4	119.91 (14)
C5—C6—C7	120.75 (14)	C16—C15—C14	120.58 (16)
C6—C7—H7A	109.5	C16—C15—H15	119.7
C6—C7—H7B	109.5	C14—C15—H15	119.7
H7A—C7—H7B	109.5	C17—C16—C15	120.24 (17)
C6—C7—H7C	109.5	C17—C16—H16	119.9
H7A—C7—H7C	109.5	C15—C16—H16	119.9
H7B—C7—H7C	109.5	C18—C17—C16	119.57 (16)
O1—C8—O2	124.49 (15)	C18—C17—H17	120.2
O1—C8—C5	126.29 (15)	C16—C17—H17	120.2
O2—C8—C5	109.17 (13)	C17—C18—C19	120.69 (18)
O2—C9—H9A	109.5	C17—C18—H18	119.7
O2—C9—H9B	109.5	C19—C18—H18	119.7
H9A—C9—H9B	109.5	C18—C19—C14	120.28 (17)
O2—C9—H9C	109.5	C18—C19—H19	119.9
H9A—C9—H9C	109.5	C14—C19—H19	119.9
H9B—C9—H9C	109.5	C6—N1—C2	118.28 (13)
C11—C10—C2	120.85 (17)	C8—O2—C9	116.98 (15)
			
N1—C2—C3—C13	179.65 (14)	C10—C11—C12—Cl1	−179.82 (14)
C10—C2—C3—C13	−0.5 (2)	C11—C12—C13—C3	−0.3 (3)
N1—C2—C3—C4	2.2 (2)	Cl1—C12—C13—C3	179.26 (12)
C10—C2—C3—C4	−177.96 (14)	C2—C3—C13—C12	0.7 (2)
C13—C3—C4—C5	−174.20 (14)	C4—C3—C13—C12	177.98 (14)
C2—C3—C4—C5	3.1 (2)	C5—C4—C14—C19	54.6 (2)
C13—C3—C4—C14	5.6 (2)	C3—C4—C14—C19	−125.18 (17)
C2—C3—C4—C14	−177.17 (14)	C5—C4—C14—C15	−124.62 (17)
C3—C4—C5—C6	−6.4 (2)	C3—C4—C14—C15	55.6 (2)
C14—C4—C5—C6	173.85 (14)	C19—C14—C15—C16	1.2 (3)
C3—C4—C5—C8	174.07 (13)	C4—C14—C15—C16	−179.56 (16)
C14—C4—C5—C8	−5.7 (2)	C14—C15—C16—C17	−0.5 (3)
C4—C5—C6—N1	4.7 (2)	C15—C16—C17—C18	−0.9 (3)
C8—C5—C6—N1	−175.70 (14)	C16—C17—C18—C19	1.6 (3)
C4—C5—C6—C7	−172.83 (15)	C17—C18—C19—C14	−0.9 (3)
C8—C5—C6—C7	6.7 (2)	C15—C14—C19—C18	−0.6 (3)
C4—C5—C8—O1	77.7 (2)	C4—C14—C19—C18	−179.76 (17)
C6—C5—C8—O1	−101.9 (2)	C5—C6—N1—C2	0.7 (2)
C4—C5—C8—O2	−104.54 (17)	C7—C6—N1—C2	178.37 (14)
C6—C5—C8—O2	75.90 (17)	C10—C2—N1—C6	176.04 (14)
N1—C2—C10—C11	179.81 (16)	C3—C2—N1—C6	−4.1 (2)
C3—C2—C10—C11	0.0 (3)	O1—C8—O2—C9	2.1 (3)
C2—C10—C11—C12	0.4 (3)	C5—C8—O2—C9	−175.75 (15)
C10—C11—C12—C13	−0.2 (3)		

### Hydrogen-bond geometry (Å, °)

**Table:**

Cg1 is the centroid of the C14–C19 ring.

**Table:**

*D*—H···*A*	*D*—H	H···*A*	*D*···*A*	*D*—H···*A*
C9—H9B···Cg1^i^	0.96	2.80	3.744 (3)	166

Symmetry codes: (i) −*x*+1/2, *y*−1/2, −*z*+1/2.
